# 24-Week Exposure to Oxidized Tyrosine Induces Hepatic Fibrosis Involving Activation of the MAPK/TGF-*β*1 Signaling Pathway in Sprague-Dawley Rats Model

**DOI:** 10.1155/2016/3123294

**Published:** 2015-12-14

**Authors:** Zhuqing Leslie Li, Yonghui Shi, Guowei Le, Yinyi Ding, Qi Zhao

**Affiliations:** ^1^The Laboratory of Food Nutrition and Functional Factors, Food Science and Technology, Jiangnan University, Wuxi 214122, China; ^2^The State Key Laboratory of Food Science and Technology, Food Science and Technology, Jiangnan University, Wuxi 214122, China

## Abstract

*Scope*. Oxidized tyrosine (O-Tyr) has been widely detected in many consumer protein products. O-Tyr products such as dityrosine (Dityr) and 3-nitrotyrosine (3-NT) are universal biomarkers of protein oxidation and have been demonstrated to be associated with metabolic disorders in biological system. Evaluation of potential intracorporal effects of dietary O-Tyr is important since the mechanism of biological impacts induced by oral oxidized protein products (OPPs) is still limited although we have proved that some dietary OPPs would induce oxidative injury to liver and kidney. *Methods and Results*. The present study aimed to investigate the dose-dependent hepatic injury caused by oral O-Tyr in rats. 24-week feeding of O-Tyr enhanced aspartate aminotransferase (AST) and alanine aminotransferase (ALT) activities, increased total bilirubin (TBiL) content, and led to oxidative damage in rats liver. Besides, O-Tyr distinctly increased the phosphorylation of p38 and ERK2 MAPKs and enhanced fibrosis-related TGF-*β*1 and Smad2/3 levels. Higher extracellular matrix (ECM) indexes (ICTP, PIIINP) and histological examination (HE and Masson staining) also supported dose-dependent hepatic fibrosis caused by O-Tyr. *Conclusion*. These findings reveal that O-Tyr may induce oxidative damage and hepatic fibrosis via MAPK/TGF-*β*1 signaling pathway, in which ROS together with malondialdehyde (MDA) and OPPs act as the pivotal mediators.

## 1. Introduction

Proteins are targets to various oxidants including radicals and nonradical oxidants. Food protein is vulnerable to be oxidized during food processing and storage, leading to structural changes such as loss of sulfhydryl groups and accumulation of protein carbonyls (PC), dityrosine (Dityr), and 3-nitrotyrosine (3-NT) [1]. Formation of oxidized protein products (OPPs) in food system has been proved to cause protein aggregation, reduce essential amino acids, and decrease protein digestibility [2–7].

In vivo studies have demonstrated that accumulation of OPPs in biological system has potential association with relevant degenerative diseases. PC, Dityr, 3-NT, and advanced oxidation protein products (AOPPs) have been widely used as biomarkers for monitoring tissue damage and evaluating a number of age-related diseases (e.g., Parkinson's disease, renal failure, diabetes, and intestinal tissue injury) [8–10]. According to the above discoveries, it is noteworthy that oral OPPs may moreover have impacts on cells and/or tissues as endogenous OPPs do if intake of dietary OPPs could induce intracorporal protein oxidation. Therefore, research on effects of dietary oxidized protein on human health has important scientific significance to avoid potential injury caused by oral OPPs.

Our past findings have shown that exposure of dietary oxidized casein leads to intestinal flora disturbance and accumulation of OPPs in mice tissues [11, 12]. We also found that oral oxidized casein containing PC, Dityr, and AOPPs impaired antioxidant defense system and induced hepatic and renal fibrosis [13]. However, the mechanism of impacts induced by oral OPPs is still limited.

Among all amino acids, Tyr is sensitive to various ROS, such as metal-catalyzed oxidants, peroxynitrite, and UV irradiation, leading to formation of Dityr, 3-NT, AOPPs, and 3-chlorotyrosine, which have been widely detected in food system and identified as universal biomarkers of protein oxidation [14, 15]. Therefore, evaluation of oral O-Tyr-induced impacts is essential for researchers to better understand the mechanism of biological impacts induced by oral oxidized protein. The objective of this study was to investigate the mechanism of O-Tyr-induced hepatic injury in a dose-dependent manner. The hepatic damage caused by O-Tyr was evaluated by measuring oxidative stress-related parameters and hepatic fibrosis biomarkers. Histological studies were performed to provide further support of hepatic injury. In addition, activation of the MAPK/TGF-*β*1 pathway was investigated.

## 2. Materials and Methods

### 2.1. Materials

Tyrosine was obtained from Sigma Chemical Co. (St. Louis, MO, USA). Anti-MAPK (p38/ERK) antibody and anti-TGF-*β* antibody were purchased from Santa Cruz Biotechnology, Inc. (Santa Cruz, CA, USA). Porcine primers were designed and synthesized by Generay Biotechnology Co. (Shanghai, China). Trizol reagent and AccuPower GreenStarTM qPCR PreMix kit were purchased from Applied Biosystems (Foster City, CA, USA). ELISA kits of dityrosine (Dityr), advanced oxidized protein products (AOPPs), aspartate aminotransferase (AST), alanine aminotransferase (ALT), total bilirubin (TBiL), cross-linked carboxy-terminal telopeptide type I collagen (ICTP), and N-terminal procollagen III propeptide (PIIINP) were obtained from Xiamen Huijia Bioengineering Institute (Xiamen, China). Detection kits for malondialdehyde (MDA), catalase (CAT), glutathione peroxidase (GPX), superoxide dismutase (SOD), and hematoxylin and eosin (HE) and Masson staining were purchased from Nanjing Jiancheng Bioengineering Institute (Nanjing, China). All other chemicals used in the experiments were of the highest quality commercially available.

### 2.2. Preparation of O-Tyr

*Tyrosine Oxidized by H*_*2*_ *O*_*2*_ *-Cu (* ^∙^ *OH).* O-Tyr was prepared by our laboratory according to the method of Kurahashi et al. [16] and Li et al. [13] with a little modification. Briefly, Tyr was dispersed in 0.05 M phosphate buffer (pH 7.4) to get samples with the concentration of 1 mg/mL. The whole Tyr solution system was mixed in sealed tubes after adding H_2_O_2_-CuSO_4_ (5 mM-0.05 mM) and was shaken at a proper speed in a thermostatic bath at 45 ± 0.1°C for 2 h. At the end of the reaction, Cu^2+^ was removed using ion exchange resin. Freeze-drying was applied to remove the remaining hydrogen peroxide and dry the O-Tyr sample.

### 2.3. Determination of Oxidized Protein Products (OPPs)

#### 2.3.1. Qualitative Analysis of Oxidized Tyrosine Products (OTPs) by HPLC-MS Chromatogram

*HPLC for OTPs Separation.* LC (Waters 1525) was performed on a Kromasil-C_18_ column (250 × 4.6 mm). The mobile phase was a gradient prepared from acetonitrile (component A) and 0.1% v/v formic acid in water (component B). The separate condition is shown in Table 1. The absorbance of the eluate at 280 nm was monitored. The sampling volume was 10 *μ*L, the flow rate was 0.8 mL/min, and the column was maintained at 45°C.

*Mass Spectrometry.* MS (Waters Platform ZMD 4000) ran in electrospray ionization mode. The optimized MS conditions were capillary voltage 3.0 kVolts, cone voltage 20 Volts, source block temperature 100°C, desolvation temperature 250°C, desolvation gas flow 500 lit/h, cone gas flow 50 lit/h, collision energy 30 eV, mass range 50 to 1500 *m*/*z*, and detector voltage 1700 Volts.

#### 2.3.2. Determination of Protein Carbonyl (PC)

PC content was determined (2,4-dinitrophenylhydrazine, DNPH) using the method of Oliver et al. [17] with slight modifications. O-Tyr samples were suspended in deionized water to provide O-Tyr solution of the concentration 3.0 to 3.5 mg/mL. In 2.0 mL capped polyethylene centrifuge tubes, 0.3 mL O-Tyr solution was mixed with 1.2 mL 10 mM DNPH in 2 N HCl and incubated at 25 ± 0.1°C for 1.0 h in the dark. A matching aliquot was mixed in 1.2 mL 2 N HCl as an absorbance blank. Then, 1.5 mL 40% trichloroacetic acid (TCA) was added to each tube. The tubes were then vortexed, allowed to stand for 20 min, and centrifuged for 15 min at a speed of 12,000 g. The supernatant was discarded, and the precipitate was washed three times with 1.0 mL ethyl acetate solution (1 : 1, v/v) and then was suspended in 1.25 mL 6 M guanidine hydrochloride solution by incubating at 37°C for 15 min, with vortexing every 5 min. After centrifugation (12,000 ×g, 15 min), the supernatant was collected and detected by the wavelength of 370 nm. The absorbance at 370 nm was corrected for the absorbance in the HCl blank, and the moles of carbonyl derivative per mg protein were calculated by using the extinction coefficient of 22,000 M^−1^ cm^−1^ [18].

### 2.4. Animal Care and Treatments

A total of 40 male Sprague-Dawley (SD) rats (4 weeks old) were obtained from Slack Shanghai Laboratory Animal Co., Ltd. (Shanghai, China). Rats were housed under conditions of controlled temperature (26 ± 2°C) and humidity (60%) with a 12 h light/dark cycle. The experimental protocol was developed according to the institution's guidelines for the care and use of laboratory animals.

After a 3-day acclimatization period, rats were randomly assigned to five groups (eight for each group): a normal diet was given to all animals for 10 days. Then, after the first group was treated with normal diet, three groups were, respectively, treated with O-Tyr added at dose of 2, 4, and 8 g/kg diet each day for 24 weeks. Moreover, we set a group of Tyr added at dose of 8 g/kg diet as Tyr control. The 24 h feed intake and body weight were measured weekly. The behavior of the animals was observed daily.

At the end of the 24-week feeding experiment, all the animals were anaesthetized using anesthetic ether. Blood samples were collected via cardiac puncture. Plasma obtained from blood samples after centrifugation (5000 rpm for 10 min at 4°C) was frozen and stored at −20°C for further measurement. In addition, organs of each rat were isolated, weighed, and dissected out for histopathological examination. Organ indexes were expressed as the organ (mg) over the body weight (g). Livers were separated on the basis of morphological features by an animal care technician and were rinsed with 0.1 mM phenylmethanesulfonyl fluoride in physiological saline and flash frozen in liquid nitrogen for further biomarker analyses.

### 2.5. Analysis of Antioxidant Enzyme Activity and Tissue Oxidation

Levels of ROS were measured in the whole blood by luminol-dependent chemiluminescence assay described by Kobayashi et al. [19], using MPI-B ultra-weak luminescence analysis system (Xi'an Remex Analysis Instrument Co. Ltd., Xi'an, China). ROS production was expressed as relative light units (RLUs). GSH and GSSG levels were determined according to the fluorimetric method of Hissin and Hilf [20] by using* O*-phthalaldehyde (OPT) as a fluorescent probe. The method takes advantage of the reaction of OPT with GSH at pH 8.0 and with GSSG at pH 12.0, resulting in a highly fluorescent derivative, which is activated at 350 nm with an emission at 420 nm. PC, Dityr, AOPPs, MDA, CAT, GPX, SOD, and total antioxidant capacity (T-AOC) were assayed using kits as described by the manufacturer's instructions. Total protein (TP) contents were determined by the method of Lowry et al. [21], using BSA as protein standard.

### 2.6. Analysis of Hepatic Injury and Fibrosis Indexes

The activities of AST, ALT and content of TBiL, ICTP, and PIIINP were determined using kits as described by the manufacturer's instructions.

### 2.7. Gene Expression Related to MAPK/TGF-*β*1 Signaling Pathway in Rats Liver

For determining mRNA expression, total RNA was first extracted from frozen tissues with Trizol reagent. The quantity and quality of the RNA were verified by measuring the *A*_260_/*A*_280_ ratio and by gel electrophoresis. Total RNA was reverse-transcribed to cDNA according to the manufacturer's instructions (MultiScribe Reverse Transcriptase, Applied Biosystems). The mRNA expression was quantified using Real-Time Polymerase Chain Reaction (RT-PCR). The primer sequences are listed in Table 2.

RT-PCR was carried out using AccuPower GreenStarTM qPCR PreMix kit on a MyiQ Single Color Real-Time PCR Detection System (7900 HT Fast, ABI) using the following conditions: 40 cycles of denaturation at 95°C for 20 s, annealing at 50°C for 30 s, and extension at 72°C for 20 s. Porcine primers were designed using Primer3 v.0.4.0 and synthesized by Generay Biotechnology Co. (Shanghai, China). The relative expression levels of the target genes were calculated as a ratio to the housekeeping gene *β*-actin.

### 2.8. Western Blot Analysis

Protein extracts were separated on 10% SDS-PAGE gel using 10 *μ*g protein per sample and then transferred onto a PVDF membrane. The membrane was blocked with 5% BSA in Tris-buffered saline (pH 7.4) containing 0.1% Tween 20. Subsequently, the blocked membrane was incubated with primary antibody including rabbit Nrf2 antibody (8882, Cell Signaling) and rabbit GAPDH antibody (8884s, Cell Signaling) at dilution of 1 : 1000 at 4°C overnight. Blots were incubated with IRDye 800CW-conjugated or 700CW-conjugated antibody (Rockland Biosciences) and infrared fluorescence images were obtained with the Odyssey infrared imaging system (Li-Cor Bioscience).

### 2.9. Histological Examination

The tissue specimens were obtained and promptly fixed in 10% phosphate-buffered formaldehyde for further studies. The specimens were embedded in paraffin, evaluated through hematoxylin and eosin (HE) stain and Masson trichrome stain, and then examined using CX31 RTSF microscope (×100) (Olympus Corporation, Tokyo, Japan).

### 2.10. Statistical Analysis

Data was reported as mean ± SD. Comparisons across groups were performed by one-way analysis of variance with post hoc Duncan's test. *P* < 0.05 and *P* < 0.01 were considered significant and highly significant difference. Analysis was done with SPSS 17.0 (SPSS, Inc., Chicago, IL, USA).

## 3. Results

### 3.1. Determination of O-Tyr Products (OTPs)

**Figure 1:**
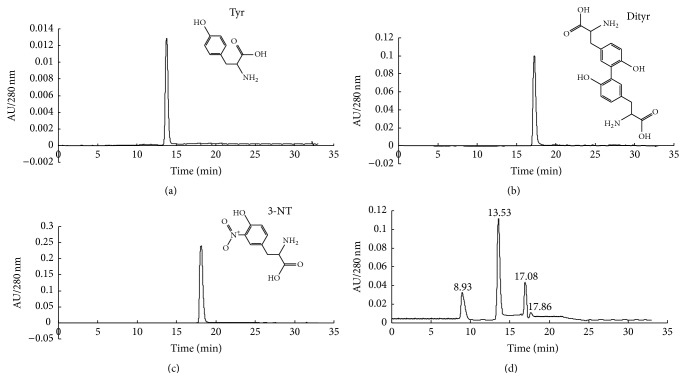
HPLC_280 nm_ chromatogram of (a) Tyr, (b) Dityr, (c) 3-NT, and (d) O-Tyr.

**Figure 2:**
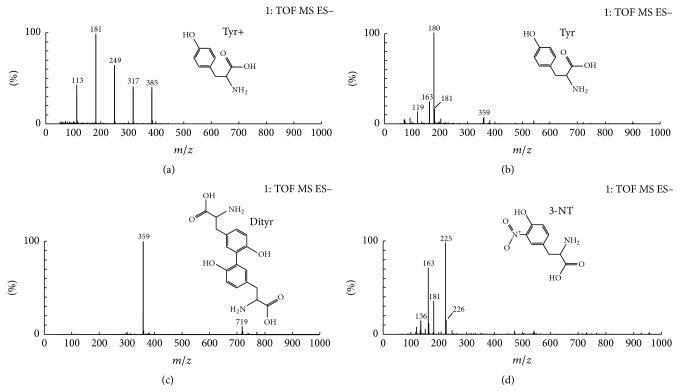
MS spectrum and structure of fractions at different retention time from HPLC_280_: (a) 8.93 min, (b) 13.53 min, (c) 17.08 min, and (d) 17.86 min.

Figure 1(d) shows HPLC results of O-Tyr which mainly contains four peaks. According to the HPLC results of standard Tyr (Figure 1(a)), Dityr (Figure 1(b)), 3-NT (Figure 1(c)), and the MS result of O-Tyr, the possible structure of each peak in Figure 1(d) (O-Tyr) is shown in Figure 2. The LC-MS results show that the O-Tyr sample used in our experiment mainly contains Tyr (Figures 2(a) and 2(b)), Dityr (Figure 2(c)), and 3-NT (Figure 2(d)).

### 3.2. Effects of O-Tyr on Body Weight and Liver Index

No mortality or abnormal clinical signs related to the administration of O-Tyr were observed. The feed intake in all the experimental groups showed no significant difference (data not shown). During the 24-week study, body weights of male rats in all O-Tyr-treated groups were less than the control (Table 3). There was no difference in absolute liver weights but a significant increased liver-to-body ratio was observed among the dose of 8 g/kg O-Tyr group (*P* < 0.05). The increased liver indices following exposure to O-Tyr may be related to hepatotoxicity and hepatic injury. Rats in the Tyr-treated group showed no significant difference versus the control animals.

### 3.3. Effects of O-Tyr on Antioxidant Capacity

**Figure 3:**
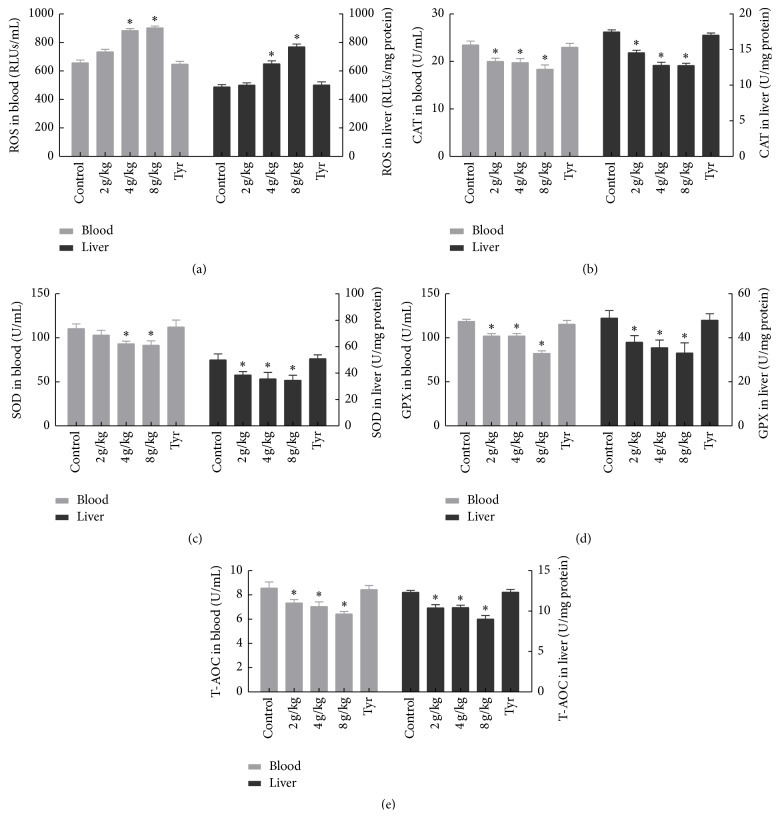
Effects of O-Tyr on (a) ROS, (b) CAT, (c) SOD, (d) GPX, and (e) T-AOC levels in blood and liver (*n* = 8).

Figure 3 gives the results of oxidative stress in blood and liver of rats. All the different doses of O-Tyr induced detectable oxidative stress on rats reflected by excess of ROS (*P* < 0.05). Compared to the control group, activities of antioxidant enzymes (CAT, SOD, and GPX) and the total antioxidant capacity (T-AOC) were all significantly limited in the O-Tyr-treated groups (*P* < 0.05). There is no difference between the control group and the Tyr group.

### 3.4. Effects of O-Tyr on Lipid Peroxidation and Protein Oxidation

**Figure 4:**
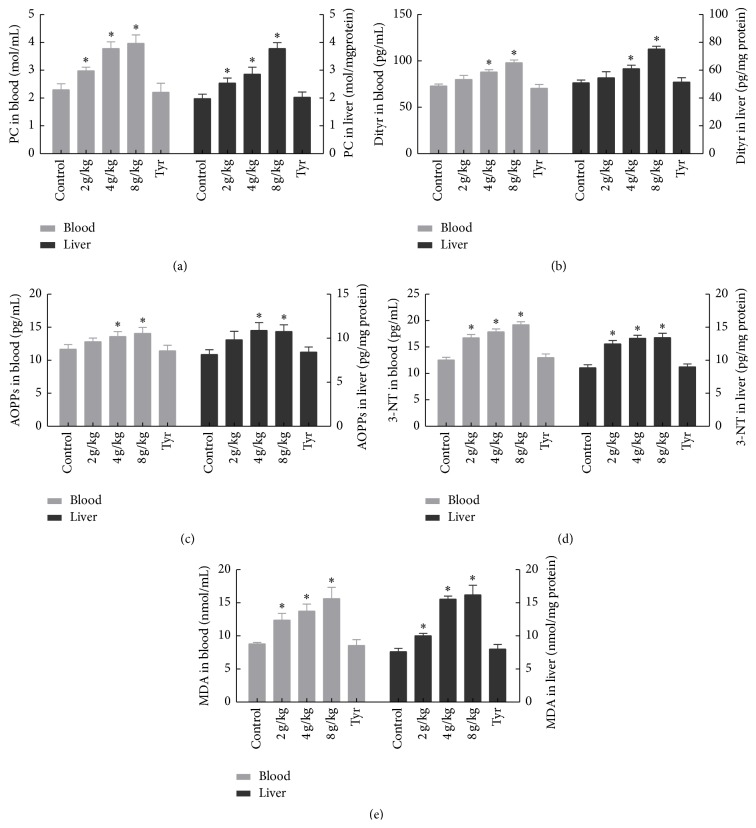
Effects of O-Tyr on (a) PC, (b) Dityr, (c) AOPPs, (d) 3-NT, and (e) MDA concentrations in blood and liver (*n* = 8).

PC, Dityr, AOPPs, 3-NT, and MDA concentrations were, respectively, determined to evaluate the oxidative damage to protein and lipids in vivo. The results (Figure 4) showed significant increases in PC, Dityr, AOPPs, 3-NT, and MDA concentrations in the 4 g/kg and 8 g/kg O-Tyr-treated groups versus the controls (*P* < 0.05). No difference was observed between the control group and the Tyr group.

### 3.5. Effects of O-Tyr on Hepatotoxicity and Hepatic Fibrosis of Rats

To confirm the effects of O-Tyr on hepatic function, serum AST, ALT, and TBiL levels were examined and shown in Table 4. The cumulative hepatotoxicity of O-Tyr was clearly featured by dose-related increase in serum AST, ALT, and TBiL levels when compared to the control (*P* < 0.05). It also can be seen that, with an increasing O-Tyr dose, levels of ICTP and PIIINP in rats liver were significantly elevated which indicates hepatic fibrosis in rats by exposure to O-Tyr. Hepatic indexes of rats in Tyr-treated group showed no difference versus the control.

### 3.6. Effects of O-Tyr on mRNA and Protein Expression Involved in MAPK/TGF-*β*1 Signaling Pathway

**Figure 5:**
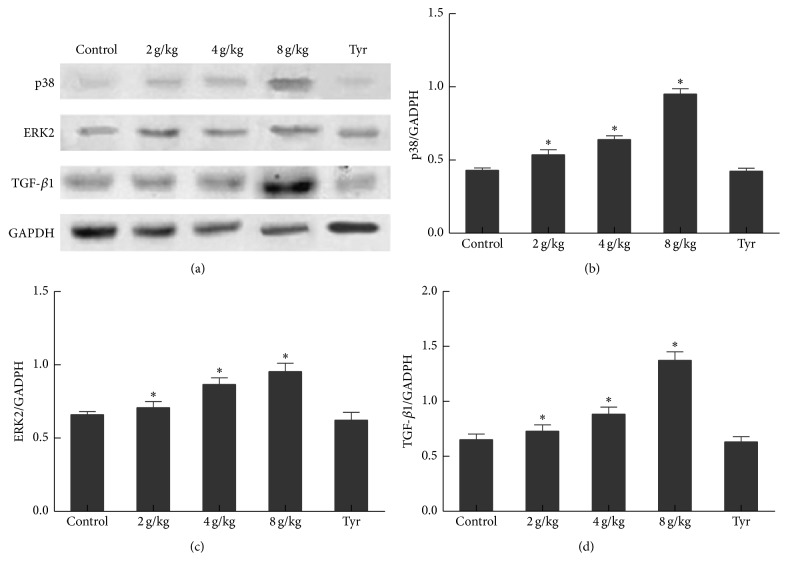
Effects of O-Tyr on protein expression in rats liver. Protein levels of (b) p38, (c) ERK2, and (d) TGF-*β*1 were measured by (a) Western Blot (*n* = 8). GAPDH was used as an internal control.

The effects of O-Tyr on the gene expression involved in MAPK/TGF-*β*1 pathway are listed in Table 5. The results suggested that O-Tyr distinctly increased the phosphorylation of p38 and ERK2 MAPKs and enhanced fibrosis-related TGF-*β*1 and Smad2/3 level (*P* < 0.05). In addition, levels of protein expression of p38, ERK2, and TGF-*β*1 all markedly increased as shown in Figure 5. There is no difference of mRNA or protein expression in the Tyr-treated group when compared to the control.

### 3.7. Histopathologic Evaluation of Hepatic Fibrosis

**Figure 6:**
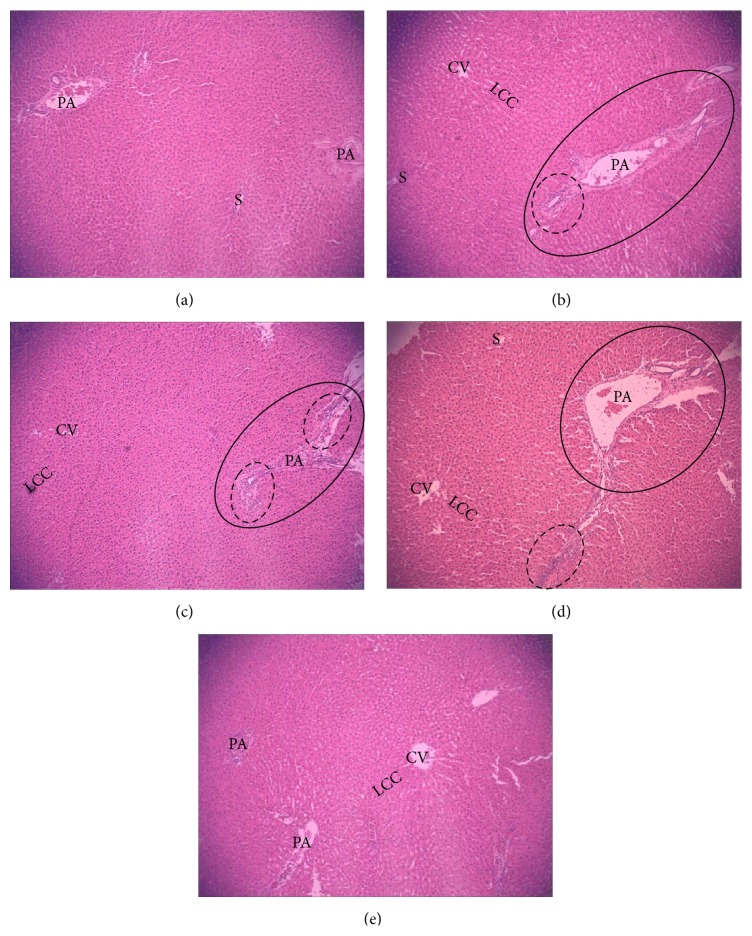
HE stain of liver sections (×100). Hepatic photomicrographs of representative rats are shown from each of the five groups. (a) Control liver tissue. (b) Liver tissue of rats treated with O-Tyr (2 mg/mg). (c) Liver tissue of rats treated with O-Tyr (4 mg/mg). (d) Liver tissue of rats treated with O-Tyr (8 mg/mg). (e) Liver tissue of rats treated with Tyr (8 mg/mg).* Notes*. CV: central vein; LCC: hepatic cell cords; S: hepatic sinusoid; PA: portal area. The dotted lines denote infiltration of inflammatory cells; the solid lines suggest tissue fibrosis in portal area.

**Figure 7:**
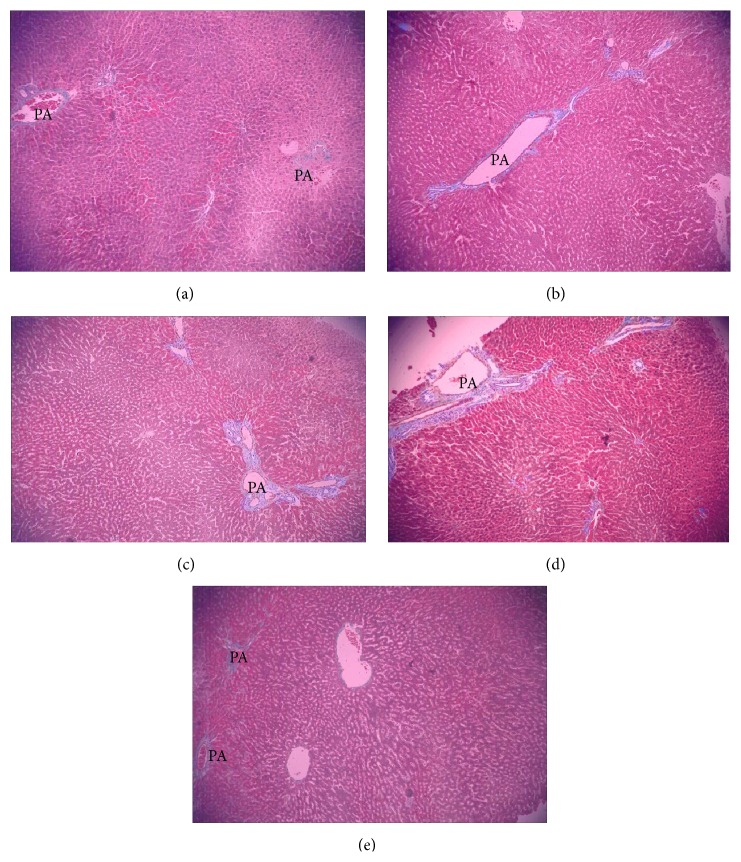
Masson stain of liver sections (×100). Hepatic photomicrographs of representative rats are shown from each of the five groups. (a) Control liver tissue. (b) Liver tissue of rats treated with O-Tyr (2 mg/mg). (c) Liver tissue of rats treated with O-Tyr (4 mg/mg). (d) Liver tissue of rats treated with O-Tyr (8 mg/mg). (e) Liver tissue of rats treated with Tyr (8 mg/mg).* Notes*. PA: portal area. The blue color suggests tissue fibrosis in portal area.

The histological changes in liver specimens by HE stain are shown in Figure 6. Under light microscope, liver samples of animals from the control (Figure 6(a)) and the Tyr-treated group (Figure 6(e)) presented regularly arranged hepatocytes and intact architecture, and there were no notable morphological alterations or fibrosis. In contrast, liver samples from rats exposed to increasing concentrations of O-Tyr exhibited severe pathological changes, including the broadened hepatic portal area, the increased fiber spread from portal area, and infiltration of inflammatory cells (Figures 6(b), 6(c), and 6(d)). Meanwhile, the Masson stain results (Figure 7) confirmed the portal and periportal fibrosis of rats in the O-Tyr groups. These results suggested that 24-week exposure to O-Tyr resulted in significant pathological changes in livers, which may be related to expression of hepatic fibrosis-related markers.

## 4. Discussion

Our previous studies indicated that exposure to oxidized casein containing PC, Dityr, AOPPs, and 3-NT resulted in oxidative stress and hepatic and renal fibrosis; however, information of the mechanism of impacts induced by oral OPPs is limited. Tyrosine (Tyr) is one of the major targets of protein oxidation, and until today various tyrosine derivatives such as Dityr, 3-NT, and halogenated Tyr were identified in food system and used as biomarkers of oxidative protein. Our O-Tyr sample mainly contains Dityr, a tyrosine dimer derived from tyrosyl radicals which has been proved to widely exist in food system, especially in meat and milk products. Fenaille et al. [22] have found that the level of Dityr ranged from below the limit of quantification to 393.0 ± 9.1 *μ*mol Dityr/mol Tyr in different milk powder samples. The study presented here used different concentrations of O-Tyr modified by ^*∙*^OH to evaluate the effects of O-Tyr on male rats.

AST, ALT, and TBiL are widely used as biochemical markers of possible liver injury. Previous studies have proved that, after hepatocellular damage, activities of AST and ALT always show higher levels in serum because of the increased membrane permeability or hepatocellular necrosis and cytosol leakage [23, 24]. High concentration of TBiL is always associated with disorder of liver metabolism [25]. The current study suggests that intake of 2, 4, and 8 g of O-Tyr/kg of diet for 24 weeks increased the activities of serum AST, ALT, and TBiL level. These changes together with a significant decrease in the body weight and higher liver index in our study indicate that long term administration of O-Tyr leads to severe hepatic toxicity. Elevation of ICTP and PIIINP levels, together with the representative images obtained from HE and Masson staining of broadened hepatic portal area, increased fiber, and infiltration of inflammatory cells support inflammatory responses and hepatic fibrosis after exposure of O-Tyr. In liver tissues, oxidative stress plays an important role in hepatic fibrosis. Our previous findings have suggested that oxidative stress is responsible for the hepatic fibrosis induced by oxidized casein [12, 13]. In this study, our results clearly reveal that O-Tyr induces detectable oxidative stress in rats reflected by excess of ROS and limited antioxidant enzyme activities (CAT, GPX, and SOD), which would result in tissue injury and inflammation [26, 27]. The accumulation of PC, Dityr, AOPPs, 3-NT, and MDA, as markers of lipid and protein oxidative injury, furthermore confirmed that rats suffered oxidative damage after 24 weeks of O-Tyr treatment. Moreover, as mentioned in previous studies, ROS, MDA, and OPPs accumulated in liver tissues would induce activation of Kupffer cells and hepatic stellate cells (HSCs), which plays an important role in hepatic fibrosis development [28–31].

Studies indicated that hepatic fibrosis is a complex pathological process that involves various cytokines and numerous cell signaling pathways [32–35]. Hepatic inflammation and fibrosis in rats following exposure to O-Tyr in our study may be due to modulation of the MAPK/TGF-*β* pathway. The excess of ROS together with MDA and OPPs in livers triggered the MAPK pathway by increasing both gene and protein expression of extracellular signal-regulated kinase (ERK2) and mitogen-activated protein kinase (p38). It has been proved that MAPK pathway is involved in hepatic fibrosis and is crucial for TGF-*β*1-stimulated HSCs [36, 37]. Transforming growth factor-*β*1 (TGF-*β*1) has been reported to trigger the activation of HSCs, leading to the generation of too much extracellular matrix and inducing their differentiation into fibroblasts [28]. TGF-*β*1 has been established as the crucial fibrogenic cytokine promoting liver fibrosis, due to its activation of HSCs via TGF-*β*1-induced phosphorylation of receptor-activated Smad2 and Smad3 [38]. The present study showed that O-Tyr activated Smad2 and Smad3 and resulted in significant increase of both gene and protein expression of TGF-*β*1. Taken together, these results suggest that O-Tyr may elevate TGF-*β*1-induced HSCs in hepatic fibrosis via activating the p38/ERK-MAPK and TGF-*β*1/Smad signaling pathways.

In addition, to further investigate whether hepatotoxicity in our experiment may partly result from the existing Tyr in O-Tyr samples, we examined a group of rats treated with Tyr (8 g/kg). Our results show that, in the Tyr group, there was no oxidative stress or hepatic inflammation in rats. Meanwhile, the liver tissues showed normal hepatocytes and there were no notable morphological alterations or fibrosis. These results proved that the hepatic injury induced by O-Tyr is mainly due to the existence of Dityr and 3-NT in O-Tyr. However, the mechanism of liver injury shown in our experiment needs more investigation.

## 5. Conclusion

In conclusion, the results obtained from the present study suggest that O-Tyr induces oxidative damage in rats. The ROS generation together with MDA and OPPs accumulation promotes hepatic fibrosis via MAPK/TGF-*β*1 pathway. Our experiments provide proof that dietary O-Tyr which mainly contains Dityr might be responsible for oxidized injury caused by oral OPPs. Our results may help researchers better understand the mechanisms of intracorporal impacts induced by oral OPPs so they could develop better methods for food protein processing. Additional work is also needed to better define the mechanisms of O-Tyr and other OPPs' metabolism.

## Supplementary Material

Graphic abstract.

## Figures and Tables

**Table 1:** HPLC condition for OTPs separation.

Time	Component A	Component B
	(acetonitrile)	(0.1% v/v formic acid in water)
0 min	5%	95%
13 min	30%	70%
23 min	80%	20%
24 min	100%	0
28 min	100%	0
32 min	5%	95%

**Table 2:** Sequences of primers used in quantitative real-time reverse transcription PCR.

Gene symbol	Forward primer (5′-3′)	Reverse primer (5′-3′)
p38	CGAGCGATACCAGAACCT	GGATTATGTCAGCCGAGTGT
ERK2	ATTTGGTCTGTGGGCTGCAT	GTCAGCGTTTGGGAACAACC
TGF-*β*1	AATTCCTGGCGTTACCT	CCTGTATTCCGTCTCCTT
Smad2	GTATGGACACAGGCTCTCCG	TGTGACGCATGGAAGGTCTC
Smad3	CGACCACCAGATGAACCACA	AATGTCTCCCCAACTCGCTG
*β*-actin	CTGAACCCTAAGGCCAACCG	GACCAGAGGCATACAGGGACAA

**Table 3:** Effects of O-Tyr on body weight (g), liver weight (g), and liver index (mg/g) (*n* = 8).

Parameters	Control	O-Tyr (2 g/kg)	O-Tyr (4 g/kg)	O-Tyr (8 g/kg)	Tyr (8 g/kg)
Initial body weight	349.56 ± 10.24	345.13 ± 16.83	340.53 ± 10.46	351.67 ± 4.18	343.20 ± 8.35
Final body weight	734.98 ± 22.45	722.64 ± 26.68	637.18 ± 20.04^*∗*^	591.88 ± 13.34^*∗*^	728.96 ± 23.51
Liver	16.78 ± 0.83	16.63 ± 0.75	15.88 ± 0.88	17.09 ± 1.02	16.03 ± 1.10
Liver index	22.83 ± 0.48	23.01 ± 0.63	24.92 ± 0.77	28.87 ± 1.16^*∗*^	23.34 ± 0.84

Notes: ^*∗*^ *P* < 0.05 and ^*∗∗*^ *P* < 0.01 versus rats in the control group, the same as Tables 4 and 5.

**Table 4:** Effects of O-Tyr on hepatotoxicity and hepatic fibrosis indexes of rats (*n* = 8).

Parameter	Control	O-Tyr (2 g/kg)	O-Tyr (4 g/kg)	O-Tyr (8 g/kg)	Tyr (8 g/kg)
Serum					
AST (U/L)	143.40 ± 5.26	151.12 ± 8.34	161.88 ± 5.60^*∗*^	168.81 ± 6.38^*∗*^	140.17 ± 8.08
ALT (U/L)	39.02 ± 2.78	43.26 ± 3.11	47.54 ± 2.08^*∗*^	52.56 ± 2.33^*∗*^	40.33 ± 3.56
TBiL (*μ*mol/L)	3.34 ± 0.18	3.90 ± 0.25	5.02 ± 0.33^*∗*^	7.13 ± 0.45^*∗∗*^	3.28 ± 0.59

Liver					
I CTP (*μ*g/mg protein)	8.34 ± 0.23	8.25 ± 0.21	9.63 ± 0.44^*∗*^	10.24 ± 0.32^*∗*^	8.11 ± 0.20
PIIINP (*μ*g/mg protein)	7.56 ± 0.34	8.62 ± 0.19^*∗*^	9.54 ± 0.33^*∗*^	10.87 ± 0.43^*∗*^	7.79 ± 0.47

**Table 5:** Effects of O-Tyr on mRNA expression fibrosis-related genes in rats liver (*n* = 8).

Genes	Control	O-Tyr (2 g/kg)	O-Tyr (4 g/kg)	O-Tyr (8 g/kg)	Tyr (8 g/kg)
p38	1.00 ± 0.23	2.34 ± 0.56^*∗*^	4.90 ± 0.53^*∗*^	6.73 ± 0.92^*∗∗*^	1.28 ± 0.34
ERK2	1.00 ± 0.23	4.56 ± 0.78^*∗*^	5.25 ± 0.42^*∗*^	8.66 ± 0.89^*∗∗*^	1.00 ± 0.27
TGF-*β*1	1.00 ± 0.34	4.72 ± 0.89^*∗*^	8.17 ± 0.94^*∗∗*^	11.01 ± 1.67^*∗∗*^	0.97 ± 0.42
Smad2	1.00 ± 0.28	3.23 ± 0.56^*∗*^	4.61 ± 0.83^*∗*^	7.55 ± 0.45^*∗∗*^	1.19 ± 0.38
Smad3	1.00 ± 0.16	4.44 ± 0.82^*∗*^	6.02 ± 0.74^*∗*^	8.57 ± 0.93^*∗∗*^	1.32 ± 0.44

## Abbreviations

AOPPs: Advanced oxidized protein products
AST: Aspartate aminotransferase
ALT: Alanine aminotransferase
Dityr: Dityrosine
GPX: Glutathione peroxidase
HE: Hematoxylin and eosin
MDA: Malondialdehyde
OPPs: Oxidized protein products
PC: Protein carbonyl
ROS: Reduced oxygen species
SOD: Superoxide dismutase
T-AOC: Total antioxidant capacity
TBiL: Total bilirubin.
