# Supplementary figures and images for: 24-Week Exposure to Oxidized Tyrosine Induces Hepatic Fibrosis Involving Activation of the MAPK/TGF-β1 Signaling Pathway in Sprague-Dawley Rats Model
Source: Oxid Med Cell Longev. 2015 Dec 14;2016:3123294. doi: 10.1155/2016/3123294 (PMC4691618; doi:10.1155/2016/3123294)

## Graphical Abstract

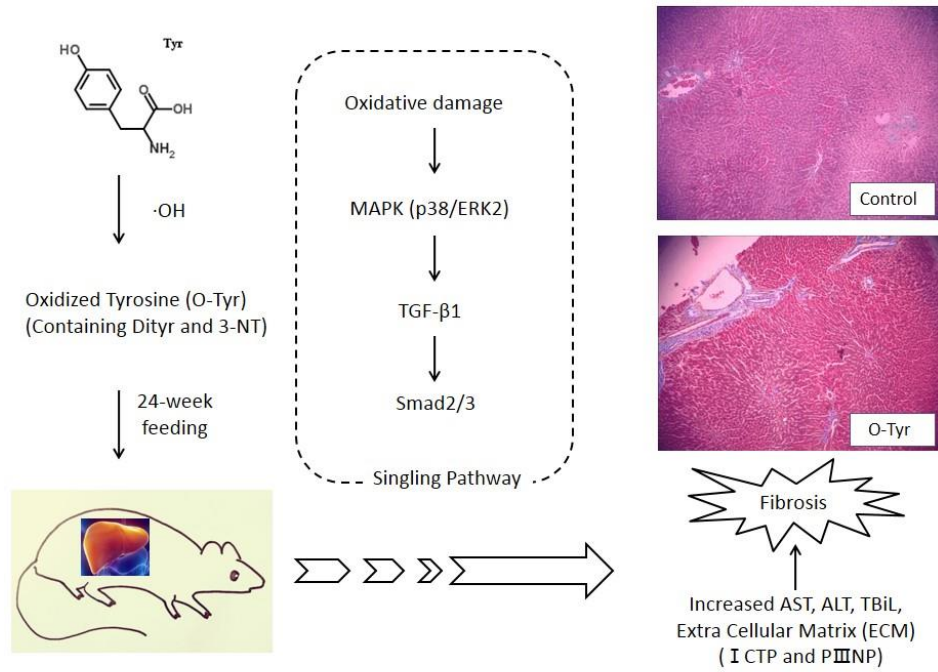

Supplement: Supplementary file 1 — Graphic abstract. [file 3123294.f1.pdf]
